# Dynamic analysis of iris changes and a deep learning system for automated angle-closure classification based on AS-OCT videos

**DOI:** 10.1186/s40662-022-00314-1

**Published:** 2022-11-05

**Authors:** Luoying Hao, Yan Hu, Yanwu Xu, Huazhu Fu, Hanpei Miao, Ce Zheng, Jiang Liu

**Affiliations:** 1 Research Institute of Trustworthy Autonomous Systems and Department of Computer Science and Engineering, Southern University of Science and Technology, Shenzhen, 518055 China; 2 Intelligent Healthcare Unit, Baidu, Beijing, China; 3 Institute of High Performance Computing, Agency for Science, Technology and Research, Singapore, Singapore; 4 School of Ophthalmology and Optometry, School of Biomedical Engineering, Wenzhou Medical University, Wenzhou, China; 5 Department of Ophthalmology, Xinhua Hospital, Shanghai Jiaotong University School of Medicine, Shanghai, China; 6 School of Computer Science, University of Birmingham, Birmingham, UK

**Keywords:** AS-OCT videos, Angle-closure, Iris change, Glaucoma, Deep learning

## Abstract

**Background:**

To study the association between dynamic iris change and primary angle-closure disease (PACD) with anterior segment optical coherence tomography (AS-OCT) videos and develop an automated deep learning system for angle-closure screening as well as validate its performance.

**Methods:**

A total of 369 AS-OCT videos (19,940 frames)—159 angle-closure subjects and 210 normal controls (two datasets using different AS-OCT capturing devices)—were included. The correlation between iris changes (pupil constriction) and PACD was analyzed based on dynamic clinical parameters (pupil diameter) under the guidance of a senior ophthalmologist. A temporal network was then developed to learn discriminative temporal features from the videos. The datasets were randomly split into training, and test sets and fivefold stratified cross-validation were used to evaluate the performance.

**Results:**

For dynamic clinical parameter evaluation, the mean velocity of pupil constriction (VPC) was significantly lower in angle-closure eyes (0.470 mm/s) than in normal eyes (0.571 mm/s) (*P* < 0.001), as was the acceleration of pupil constriction (APC, 3.512 mm/s^2^ *vs.* 5.256 mm/s^2^; *P* < 0.001). For our temporal network, the areas under the curve of the system using AS-OCT images, original AS-OCT videos, and aligned AS-OCT videos were 0.766 (95% CI: 0.610–0.923) *vs.* 0.820 (95% CI: 0.680–0.961) *vs.* 0.905 (95% CI: 0.802–1.000) (for Casia dataset) and 0.767 (95% CI: 0.620–0.914) *vs.* 0.837 (95% CI: 0.713–0.961) *vs.* 0.919 (95% CI: 0.831–1.000) (for Zeiss dataset).

**Conclusions:**

The results showed, comparatively, that the iris of angle-closure eyes stretches less in response to illumination than in normal eyes. Furthermore, the dynamic feature of iris motion could assist in angle-closure classification.

**Supplementary Information:**

The online version contains supplementary material available at 10.1186/s40662-022-00314-1.

## Background

Glaucoma is an eye disease with extremely complex etiology, ranking second among the four major blinding eye diseases. Globally, due to the aging of the population, the number of glaucoma patients (40–80 years old) is increasing every year. By 2040, it is estimated that 112 million people in the world will be affected by this disease [1, 2]. For Asians, primary angle-closure glaucoma (PACG) is more prevalent, which is defined as appositional or synechia closure of the anterior chamber angle and can lead to aqueous outflow obstruction and raised intraocular pressure (IOP) with glaucomatous optic neuropathy [2]. Fortunately, it can be observed by anterior segment optical coherence tomography (AS-OCT), which is a fast, efficient, and non-contact in vivo imaging device. It is used for quantitative measurement of anterior chamber structural parameters such as anterior segment angle (ACA), iris, cornea, etc. [3]. Therefore, the high prevalence of PACG in Asia underlies the need for an effective screening tool (such as AS-OCT) for early screening of the disease.

The major mechanism implicated in PACG is pupillary block, which is characterized by increased resistance to aqueous flow from the posterior to the anterior chamber at the level of the lens-iris interface [4]. There are several pieces of research to explore the association between PACG and static anatomical parameters such as the depth of the anterior segment in AS-OCT images [5–7]. However, the differences in iridopupillary dynamics may play a role in the pathogenesis of angle closure [8], and thus the effect of pupillary block can be presented more discriminatively and fully when the iris of the anterior segment is in dynamic motion. Quigley et al. [9] found that the decrease of iris cross-sectional area with pupil enlargement may be a risk factor for angle closure. Narayanaswamy et al. [10] observed changes of the iris volume under physiological dilation for several types of PACG and found that the iris volume of chronic angle-closure glaucoma is decreased, while that of the contralateral eye of acute angle-closure glaucoma is almost unchanged. Comparable results are also reported in southern India: iris area and iris volume decrease in normal eyes and primary angle-closure eyes with dilated pupil, but the reduction of iris volume in primary angle-closure eyes is significantly lower than that in normal eyes [11].

Besides, Seager et al. [12] found that the baseline iris area under dark conditions in the Chinese population is smaller than that in European and African populations, but the decrease of iris area with pupil dilation is smaller than that in the latter, which may contribute to the high prevalence of acute angle-closure in China. In vivo imaging of iris cross-sectional area showed that there is no significant change in iris volume after iris dilation for patients with PACG [9, 13]. This phenomenon indicated that the iris is spongy and compressible in the eyes of healthy and primary open-angle glaucoma (POAG) subjects, but it is incompressible in the eyes of PACG and suspected angle-closure subjects [14]. Lifton et al. [15] found that beneficial angle widening effects of transitioning from dark to light are attenuated in eyes with primary angle-closure disease (PACD), which appears related to aberrant dark to light change in anterior chamber width (ACW). To further verify their correlation, researchers studied the movement features of angle-closure eyes and angle-opening eyes under standard and dark conditions. Results showed the angle-closure group has a slower iris contraction speed in the reflection of light, which becomes faster after receiving laser peripheral iridotomy treatment [8, 16, 17].

All the above studies found that pupil constriction is an independent risk factor for PACG. Therefore, in this paper, we further studied the correlation between dynamic clinical parameters and PACG, adopting the iris movement videos captured in real-time video recordings of AS-OCT under changing illumination conditions, for early PACG screening. Moreover, for automatically extracting dynamic discriminative features to recognize angle-closure eyes, a deep learning-based temporal classifier network was proposed to detect angle-closure status using the AS-OCT video datasets.

Although several angle-closure classification methods are based on the sequence dataset collected in both dark and bright illumination conditions [18–20], the datasets and methods are completely different from ours. For datasets, the videos in our datasets capture the iris motion when illumination changes over time, while the datasets in those studies [18–20] are collected at a single moment under a dark or bright condition without any dynamic information. For methods, the deep network used in this paper focuses on learning the temporal information of iris dynamics over time, while the methods used in previous works mainly learn the spatial information of static or sequence images simultaneously, which ignore the dynamic information. Our datasets and corresponding methods are also innovative and have not been used/studied in previous works.

## Methods

### Study design and participants

Subjects were recruited from the outpatient and inpatient departments of Joint Shantou International Eye Centre of Shantou University and the Chinese University of Hong Kong, which included patients and volunteers over the age of 40 years. The study conducted adhered to the tenets of the Declaration of Helsinki and had the approval of the institutional review board (EC 20121123(5)-P15 and SUMC2013XM-0070). Written informed consent was obtained from all subjects.

All enrolled subjects were stratified into two categories: angle-closure and normal. The normal group consisted of age-related cataract patients and normal volunteers. A detailed medical history was collected for all patients, and they each underwent an ophthalmological examination. Details of the groups are as follows:

1. Angle-closure group: a glaucoma specialist (CZ) performed the gonioscopy exam in a dark room by using a Goldmann 2-mirror lens (Haag-Streit AG, Bern, Switzerland) at 16 × magnification. The angle was graded using the modified Shaffer grading system (grade 0 = no structures visible; grade 1 = Schwalbe’s line visible; grade 2 = anterior trabecular meshwork visible; grade 3 = posterior trabecular meshwork or scleral spur visible; grade 4 = ciliary body visible). Angle-closure was defined as three or more quadrants in which pigmented trabecular meshwork could not be visualized [21]. More specifics [8, 21–24] are provided in the supplemental material.
2. Normal group: Subjects who had IOP ≤ 21 mmHg, normal optic nerve, normal visual field, and those with a family history of glaucoma were included. In the current study, we defined the normal visual field as follows: a mean deviation (MD) and pattern standard deviation (PSD) within 95% confidence limits and a glaucoma hemifield test (GHT) result within normal limits [25].

Additionally, subjects were excluded if they: (1) have a history of intraocular surgery and ocular trauma; (2) have a disease or have taken systemic drugs which affect light reflection; (3) have pterygium; (4) cannot cooperate during the examination.

### AS-OCT videos acquisition

Our AS-OCT video datasets were collected by two devices: swept-source OCT [26] (Casia SS-1000 OCT, Tomey, Nagoya, Japan) and Visante OCT [27] (Visante OCT, Model 1000, software version 2.1; Carl Zeiss Meditec).

The Casia swept-source OCT is a Fourier-domain swept-source OCT designed specifically for imaging the anterior segment. With a substantial improvement in scan speed (30,000 A-scans per second), the anterior chamber angles can be imaged in 128 cross-sections (each with 512 A-scans) 360° around the anterior segment in 2.4 s [26]. For our dataset collection, the frame rate is set to 8.

The Zeiss Visante OCT is a noncontact optical coherence tomographic system that uses 1310 nm wavelength light to capture high-resolution cross-sectional images of the ocular anterior segment [27]. It allows real-time imaging of the anterior chamber with a scan speed of 2000 A-scans per second. The scan acquisition time is 0.125 s per line for the anterior segment single scan (limbus to limbus; eight frames per second). Video-recording software (Camtasia 6.0; TechSmith Corporation, Okemos, MI) at the default recording rate of 14 frames per second was utilized to capture the iris dynamic changes in response to dark-light illumination.

The process of AS-OCT video acquisition was the same for both devices. Specifically, recording of the AS-OCT videos began one minute after dark adaption using a standard protocol. The eye was then illuminated by a pen torchlight (approximately 1700 lx). The pupil and anterior chamber changes from dilatation in the dark to constriction in the light were recorded. A single ophthalmologist performed all AS-OCT testing. The process was repeated if any abnormal eye movement occurred.

### AS-OCT video datasets

For each video, the ground-truth label of normal or angle-closure was determined from the majority diagnosis of a senior ophthalmologist. For the Casia dataset, all the videos were captured along the eye’s optical axis by the swept-source OCT. The whole dataset consisted of 175 videos, including 94 videos of normal eyes and 81 videos of eyes with angle closure. The resolution of video frames was 1644 × 1000. For the Zeiss dataset, all the videos were captured by the Visante OCT. The whole dataset consisted of 194 videos, including 116 videos of normal eyes and 78 videos of eyes with angle closure. The resolution of video frames was 600 × 300. The size of the video frames to be entered into the deep learning network were fixed at 224 × 224.

### AS-OCT image dataset

To prove the superiority of classification based on the AS-OCT videos, we compared our framework with the present algorithm based on single AS-OCT images. We selected frames from the beginning and end of our videos taken in a dark environment which is the same as the present classification algorithms' datasets. For the Casia image dataset, the selected images were combined into a new training set with a total of 2680 AS-OCT images (1480 angle-closure and 1200 normal images) with the same distribution as the video dataset. For the Zeiss image dataset, it included 3880 AS-OCT images (1560 angle-closure and 2320 normal images).

### Measurements of velocity and acceleration of iris movement

**Fig. 1:**
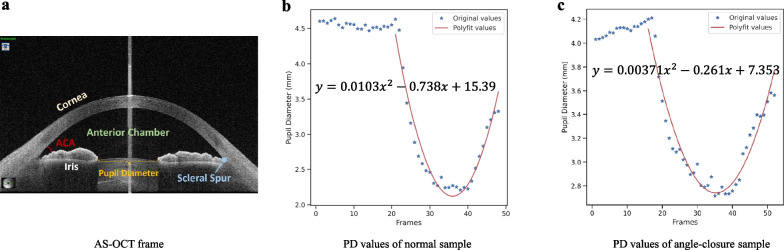
Illustration of a video frame and pupil diameter (PD) changing trends for normal and angle-closure samples. **a** Illustration of an anterior segment optical coherence tomography (AS-OCT) video frame; **b** PD value changes of a normal AS-OCT video with the frame sequence; **c** PD value changes of an angle-closure AS-OCT video with the frame sequence. The fitted trend line (red curve) is a second-order polynomial function that is denoted on the chart

To measure iris changes in response to illumination using angle-closure and normal AS-OCT videos, this study adopted the velocity and acceleration of pupil diameter (PD) over time (Fig. 1). The changes of PD could reflect the motion of the iris directly. For improved evaluation of the pupil contraction’s association with angle closure, we utilized five parameters to describe the dynamic features: maximum acceleration of pupil constriction (APC_max_), fitting acceleration of pupil constriction (APC_fitting_), average acceleration of pupil constriction (APC_mean_), maximum velocity of pupil constriction (VPC_max_) and average velocity of pupil constriction (VPC_mean_).

Here, we marked the pupillary margin manually under the guidance of a senior ophthalmologist, which is the inner border of the iris, delineating the pupil [28]. For each video sample, we calculated the transient velocities and accelerations with adjacent frames and then got the APC_max_, APC_mean_, VPC_max,_ and VPC_mean_. To obtain the iris dynamic parameters from the whole moving process, we calculated the fitting acceleration of pupil constriction modeling the curve of motion. Since changes in the PD were nonlinear over the time series (frame sequences) between the onset and the end of pupil contraction [23], a standard second-order polynomial (or quadratic function) could be fitted to the data over a video as shown in Fig. 1. The fitting model was defined as follows:

$${y}_{pd}=a{x}^{2}+bx+c$$

where $a$, $b$ and $c$ are the coefficients of the quadratic function. According to the kinematics model definition, the ${APC}_{fitting}$ is equivalent to $2a$ (mm/s^2^), as:

$${APC}_{fitting}=2a$$

It was worth noting that the curve opening was different in size for both videos, specifically, for the curve corresponding to the normal eye which was narrower. Changes of PD in the normal eye were shown to be faster.

### Angle-closure classification system

Due to the impact of involuntary eye movement and improper placement of the optical axis of the eye, the misalignments existing between consecutive video frames may contain movements of the cornea, resulting in the video frame sequence being unreliable which greatly affects the classification validation for iris motion [29]. To reduce video jitter, under the guidance of a senior ophthalmologist, we marked the corner of the ACA for all video frames and aligned the consecutive frames with the marked points. Specifically, we first calculated the rotation and translation vectors based on the marked points between frame A (the first frame of a video) and frame B (another video frame). Then, the vectors would tell us how much frame B needed to be translated and rotated to align with frame A. Based on the vectors, frame B can be transformed to the position of frame A. By repeating these steps for all video frames, the ACAs for one video could be aligned to the same position.

**Fig. 2:**
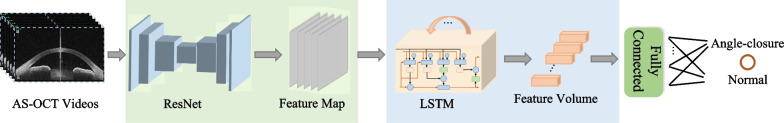
The pipeline of our angle-closure classification system

To illustrate the auxiliary effect of dynamic information on angle-closure classification, we first learned features from AS-OCT images in the videos frame by frame with ResNet [30], then long short-term memory (LSTM) [31] layers were employed to extract temporal information from video frame sequences. ResNet has a strong ability to extract image features and reduce the amount of computation during training. The main difference between the LSTM network and convolutional neural network (CNN) is that LSTM can keep information continuously by encoding state and modeling the long-term dependencies between the feature graphs along the time axis. In this paper, a LSTM layer with batch normalization and 512 hidden units [32] was positioned after the last average pooling layer of a ResNet model. We then added a fully connected layer on top of the output of the LSTM to perform multi-class classification [33], as shown in Fig. 2.

## Results

Our experiments were divided into two parts to evaluate iris changes in response to illumination. First, we calculated the five dynamic parameters using AS-OCT videos in angle-closure and normal eyes and evaluated their association with angle closure. Then, an angle-closure classification system was proposed to classify angle-closure states by modeling both motion and appearance changes in the AS-OCT video datasets.

### Dynamic parameter analysis

**Fig. 3:**
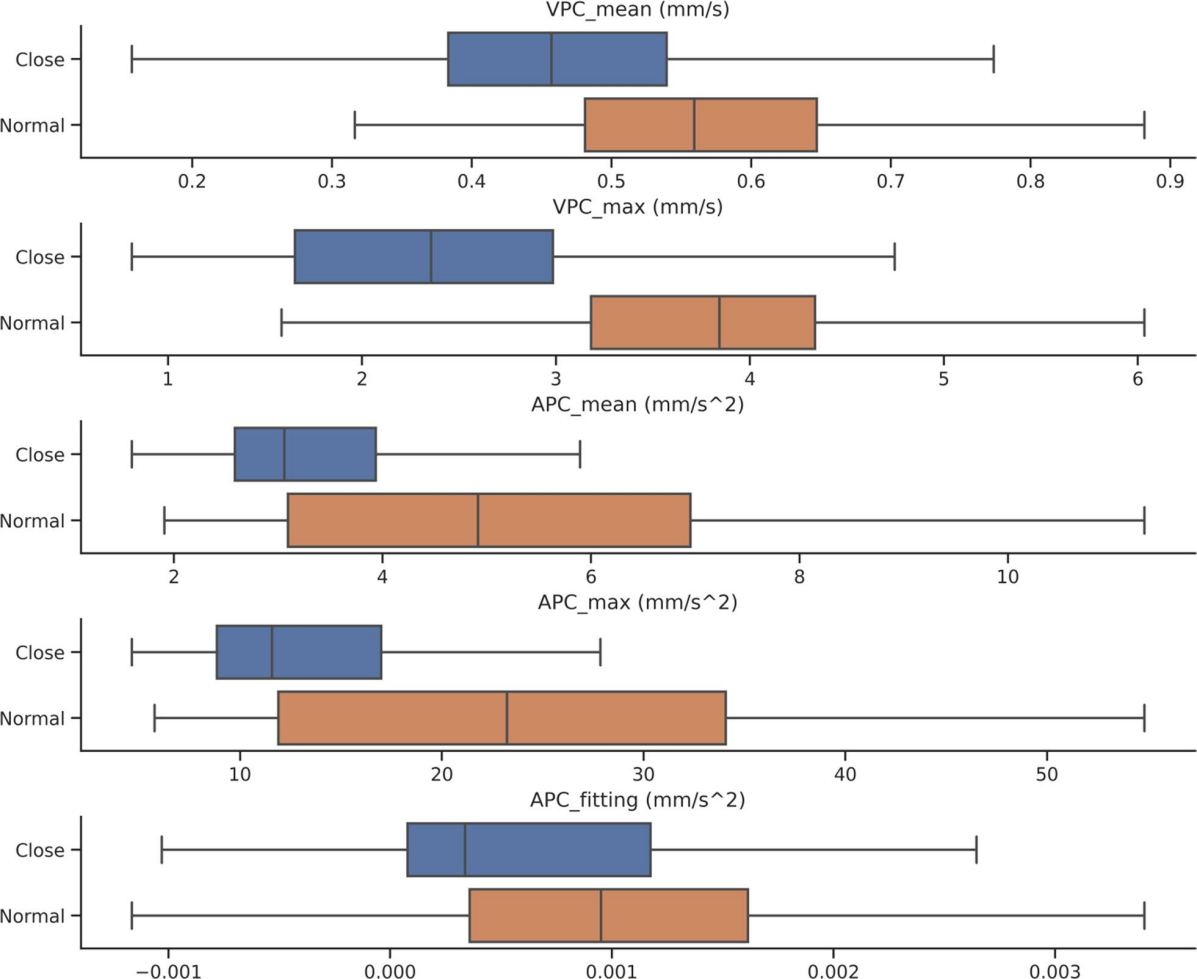
The distribution of dynamic parameters for each groups’ pupil diameter (PD). VPC, velocity of pupil constriction; APC, acceleration of pupil constriction

To reflect the difference of the iris changes in response to illumination with more samples, we combined the two datasets to build a new angle-closure group and normal angle group. Thus, we employed a dataset including 369 videos with 210 videos of normal eyes and 159 videos of eyes with angle closure. The distributions of the five parameters for the normal and angle-closure groups are shown in Fig. 3, where the black vertical line in each box represents the median of the parameter. It was noted that there was an obvious difference in the medians for velocity and acceleration of pupil constriction between the two groups.

To further illustrate the statistical significance of the difference in data distributions, the independent samples t-test was employed to examine differences in mean values of parametric data among eyes of different groups. Table 1 shows the gap of the mean value of five parameter distributions between normal- and closure-angle groups. During pupil constriction, all the VPC_mean_ (0.470 mm/s *vs.* 0.571 mm/s), VPC_max_ (2.414 mm/s *vs*. 3.802 mm/s), APC_max_ (14.376 mm/s^2^ *vs*. 23.188 mm/s^2^), APC_mean_ (3.512 mm/s^2^ *vs*. 5.256 mm/s^2^), and APC_fitting_ (0.0007 mm/s^2^ *vs*. 0.0012 mm/s^2^) were slower in the angle-closure group than in the normal group, and the differences were statistically significant (*P* < 0.001). Sample size estimation was also conducted in this study. We perfomed post hoc tests as they provide critical information about sample sizes to determine whether the sample size is enough to detect statistically significant and clinically meaningful differences between different treatment groups [34]. The power of the current study for the five parameters was 0.967–1.000 (high power), as shown in Table 1. All parameters showed good power in our study, and these features had differences between the two groups with the current sample size.

**Table 1:** Comparison of the mean values and standard deviation (SD) of dynamic parameters between normal and closed-angle groups

Parameter	Angle-closure, mean (SD)	Normal, mean (SD)	*P* value	Power
VPC_mean_ (mm/s)	0.4704 (0.1362)	0.5709 (0.1296)	< 0.001	1.0000
VPC_max_ (mm/s)	2.4143 (1.0586)	3.8020 (1.0121)	< 0.001	1.0000
APC_max_ (mm/s^2^)	14.3758 (8.3453)	23.1878 (12.1207)	< 0.001	1.0000
APC_mean_ (mm/s^2^)	3.5118 (1.5416)	5.2561 (2.4205)	< 0.001	1.0000
APC_fitting_ (mm/s^2^)	0.0007 (0.0011)	0.0012 (0.0014)	< 0.001	0.9968

*APC*_*max*_ = maximum acceleration of pupil constriction; *APC*_*fitting*_ = fitting acceleration of pupil constriction; *APC*_*mean*_ = average acceleration of pupil constriction; *VPC*_*max*_ = maximum velocity of pupil constriction; *VPC*_*mean*_ = average velocity of pupil constriction

### Classification results comparison

**Fig. 4:**
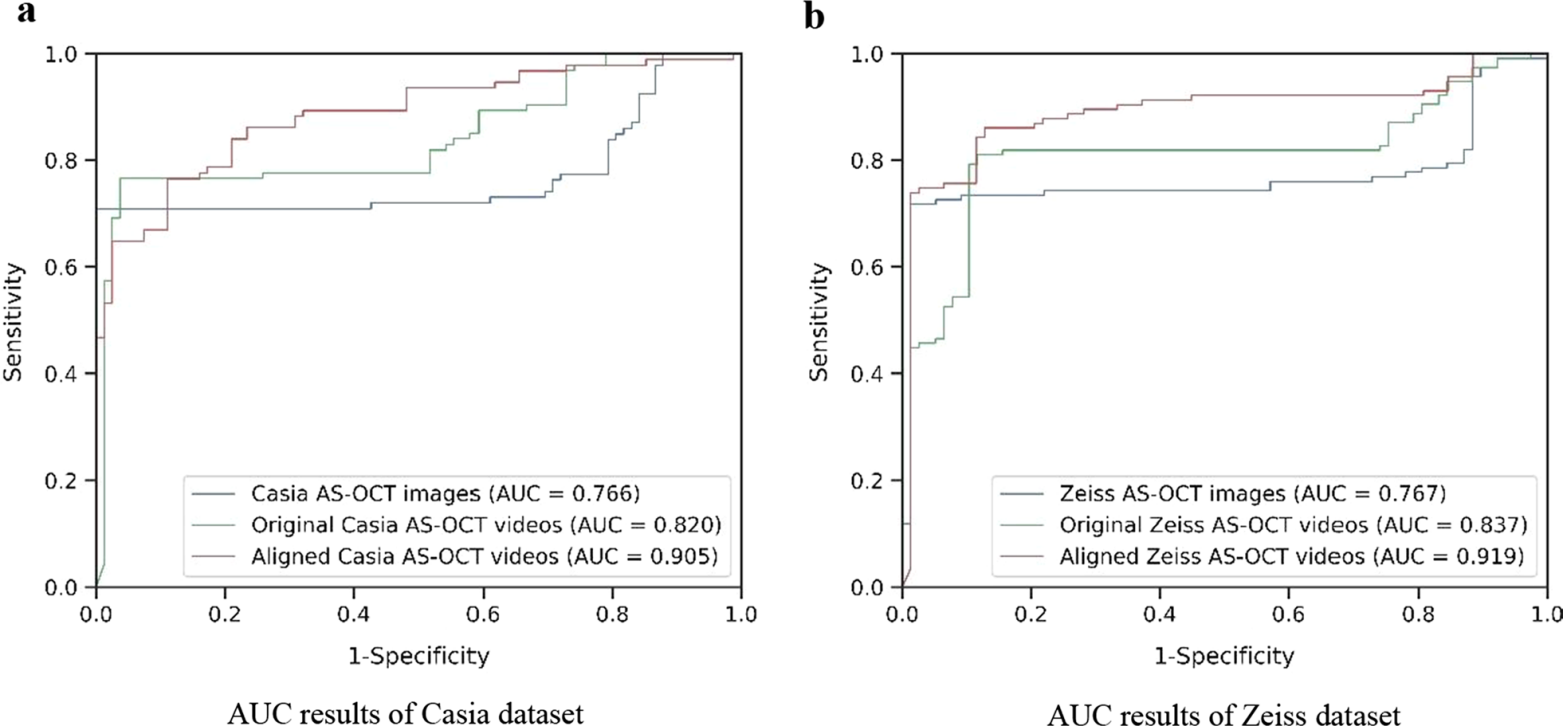
Classification results of normal and closed angles for the two datasets. **a** Area under the receiver operating characteristic curve (AUC) results of Casia dataset; **b** AUC results of Zeiss dataset. The classifications of normal and closed angles for the two datasets are under different scenarios: extracted anterior segment optical coherence tomography (AS-OCT) images, with and without video alignment over two AS-OCT video datasets

Following the clinical parameter analysis, angle-closure and normal samples can be distinguished by velocity and acceleration of pupil constriction, the ResNet-LSTM model can leverage temporal information to predict the binary classification (angle status) result. Here, we used fivefold stratified cross-validation on our two AS-OCT video datasets to evaluate the performance, which is a resampling procedure widely used to evaluate machine learning models on a limited data sample. In our study, each dataset was randomly split into five individual groups. One group was then taken as a test dataset to evaluate performance, while the other four groups were used as a training set to train the model. Finally, the cross-validation process was repeated five times, with each of the subsamples used exactly once as the test data, to report the average performance in our study [35], as shown in Table 2 and Fig. 4. We classified the angle-closure eyes by video. To measure the performance of our network more comprehensively, we employed eight evaluation criteria: area under the receiver operating characteristic curve (AUC), balanced accuracy, precision, recall, F1 score, sensitivity, specificity, and Kappa analysis. Kappa analysis and F1 score were used to reflect the trade-offs between sensitivity and specificity. We can see that there is a gap for the specificity values of the two datasets, which is due to the distribution of our datasets. The number of normal videos is 16% more than the angle-closure videos for the Casia dataset, while it is 48.7% for the Zeiss dataset. For the Zeiss dataset, our network can be better trained by normal subjects and captured the temporal feature better compared with the Casia dataset. Therefore, the Zeiss dataset demonstrates better specificity values.

**Table 2:** Comparison of the classification performance on private anterior segment optical coherence tomography (AS-OCT) video datasets and image datasets

	AUC (95% CI)	Sensitivity	Specificity	Accuracy	Precision	Recall	F1 Score	Kappa
Casia dataset								
Images	0.766 (0.610–0.923)	0.790	0.750	0.771	0.771	0.771	0.771	0.539
Original videos	0.820 (0.680–0.961)	0.833	**0.765**	0.800	0.801	0.800	0.800	0.599
Aligned videos	**0.905 (0.802–1.000)**	**0.947**	0.690	**0.829**	**0.844**	**0.829**	**0.824**	**0.648**
Zeiss dataset								
Images	0.767 (0.620–0.914)	0.750	0.933	0.821	0.852	0.821	0.823	0.643
Original videos	0.837 (0.713–0.961)	0.826	0.813	0.821	0.823	0.821	0.821	0.633
Aligned videos	**0.919 (0.831–1.000)**	**0.913**	**0.933**	**0.921**	**0.923**	**0.921**	**0.921**	**0.837**

*AUC* = area under the receiver operating characteristic curve; *CI* = confidence interval

The bold numbers indicate optimal results

AUC provides an aggregate measure of performance across all possible classification thresholds. Performances across many different thresholds were summarized with an AUC with 95% confidence intervals (CIs) as shown in Fig. 4 and Table 2. For the Casia AS-OCT video dataset, the AUC for angle-closure detection using the temporal network was 0.820 (95% CI: 0.680–0.961) with a sensitivity of 0.833 and a specificity of 0.765. Correspondingly, after video alignment, the temporal network obtained better performance, with AUC 0.905 (CI 95%: 0.802–1.000) with a sensitivity of 0.947 and a specificity of 0.690. The performance was similar for the Zeiss AS-OCT dataset, the AUC for angle-closure detection using temporal network was 0.837 (95% CI: 0.713–0.961) with a sensitivity of 0.826 and a specificity of 0.813. Correspondingly, after video alignment, we obtained superior performance, with AUC 0.919 (CI 95%: 0.831–1.000) with a sensitivity of 0.913 and a specificity of 0.933.

To compare the classification performance with the extracted AS-OCT image dataset (more details about the AS-OCT image dataset are provided in supplemental material), we employed ResNet, which was consistent with the feature extraction network in our temporal network. The results are shown in Table 2, in which the bold numbers indicate optimal results. Our network-based on AS-OCT videos gave better evaluation metrics with an obvious gap.

The classification results illustrated the importance of global change in the iris regions for improving classification performance. The performance significantly improved after the video was aligned, which was mainly because of the negative effect of video jitter on the extraction of iris dynamic features. By comparing with the performance based on AS-OCT images, it also demonstrated that the feature of iris dynamic movement under the dark-light-dark environment was helpful for the angle-closure state classification.

## Discussion

Several methods have recently been proposed to identify the normal or closed status of ACA from AS-OCT images based on anterior chamber depth. Some researchers proposed computer-aided angle-closure screening algorithms to reduce doctors’ burdens [18–20, 36–39]. Nongpiur et al. [40] proposed a classification algorithm based on stepwise logistic regression, which combined six parameters obtained from AS-OCT horizontal scan to identify angle-closure subjects; CNN was also used in this task. Fu et al. used a sliding window to detect the ACA region, and then proposed a multi-level deep network combined with global and local layer feature representation to detect the angle status of AS-OCT images [5, 41].

**Fig. 5:**
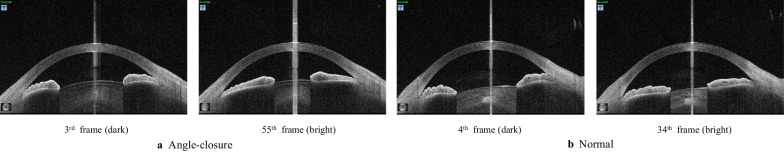
The example of (**a**) angle-closure and (**b**) normal video

However, static anatomical factors alone cannot fully explain the high prevalence of PACG, while dynamic changes of anterior chamber structure are more convincing for the diagnosis [42]. For example, Fig. 5a is an angle-closure video sample with the angle status in dark (3rd frame) and bright condition (55th frame), while Fig. 5b shows a normal sample with the angle status in dark (4th frame) and bright condition (34th frame). For the two samples, it was noted that the ACA status was almost closed in dark environments, but after light illumination, the angles become larger. For example, videos such as Fig. 5 account for about 20.6% of the angle-closure samples and 22.5% of the normal samples in our Casia dataset. Thus, it may lead to inconsistent results for the same sample if only based on a single image.

An increasing number of studies suggest that iris dynamic differences were associated with closed angles [8, 23, 27, 28, 43, 44]. There are also several angle-closure classification methods using datasets collected under both dark and bright illumination conditions [18–20]. Hao et al. [18, 19] proposed a multi-sequence deep network, which learned to identify discriminative representations from a sequence of AS-OCT images. Li et al. [20] developed a three-dimensional deep learning-based automated digital gonioscopy system in detecting narrow iridocorneal angles and peripheral anterior synechiae in eyes with suspected PACG. However, the datasets they used do not contain the temporal information of iris dynamic changes over time. Instead, for our dataset, the pupil and anterior chamber changes from dilatation in the dark to constriction in the light were recorded. Although some studies analyze iris dynamic differences for angle-closure subjects [8, 23, 27, 28, 43, 44], they are mainly based on traditional image processing and measurement of relevant anatomical parameters, and our proposed method is the first end-to-end deep learning classification on AS-OCT videos. Based on the findings [8, 23] regarding iris change in response to illumination for angle-closure subjects, we further evaluated it from two different perspectives based on two datasets. We first calculated five dynamic parameters of pupil constriction during dark-light illumination change. The results showed that angle-closure eyes have a significantly slower speed and smaller acceleration of pupil constriction in response to light compared to normal eyes. Furthermore, the deep learning method learned the highly discriminative temporal representations from the AS-OCT videos. The experiments demonstrated that the deep learning method enables automated identification of angle closure with a high AUC score.

One limitation of this study is that a specific Asian population (Chinese) was evaluated, and the results may not apply to other ethnic groups. Second, we only analyzed the scans from the nasal side to the temporal side to avoid occlusion of eyelids. The data measured from a single direction cannot perfectly express the overall state of the anterior segment. Third, the current sample size for data analysis was small, which therefore limits what can be achieved using deep learning. We believe that the model should become stable and more powerful as the sample size increases. Fourth, the AS-OCT videos were taken from our devices, this could negatively affect the quality and performance when the network is applied to videos from other AS-OCT acquisition devices. In addition, we validated our temporal network by separating the devices. The effectiveness of our network is not evaluated on more devices or multi-center datasets. Fifth, the pupil dynamics of other glaucoma subjects, like open-angle glaucoma, have not yet been assessed; the pattern of pupil dynamics may be different in other types of glaucoma. Finally, since the main focus of this study was the real-time motion parameters in the iris and its relationship with angle closure, other factors related to angle closure, such as iris surface features, iris volume, and iris curvature, were not studied.

## Conclusions

In summary, after measuring velocity and acceleration of pupil contraction in response to dark-light changes using AS-OCT videos and evaluating their association with angle closure, we further developed a deep learning framework to learn discriminative temporal features from AS-OCT videos. Deep learning is a promising technology for helping clinicians in reliably identifying angle closure in AS-OCT videos with high AUC scores. The proposed framework opens the possibility for further enhancing the ability of angle-closure related disease screening from a new perspective. Additional studies are required to explore the utility of deep learning algorithms deployed in different population settings, with the use of multiple manufacturers’ devices and larger AS-OCT datasets. In our future work, we will focus on creating a larger, multi-device and multi-center dataset. We will also assign more importance to developing multi-device compatibility/fusion algorithms and continuously improve the network’s generalization, robustness, and accuracy.

## Supplementary Information

**Additional file 1.** The specific categories for the subjects of the angle-closure group.

## Data Availability

The datasets generated and/or analyzed here are available upon reasonable request from the corresponding author.

## Abbreviations

PACG: Primary angle-closure glaucoma
AS-OCT: Anterior segment optical coherence tomography
ACA: Anterior segment angle
POAG: Primary open-angle glaucoma
PACS: Primary angle-closure suspect
PAC: Primary angle-closure
APAC: Acute primary angle-closure
APC_max_: Maximum acceleration of pupil constriction
APC_fitting_: Fitting acceleration of pupil constriction
APC_mean_: Average acceleration of pupil constriction
VPC_max_: Maximum velocity of pupil constriction
VPC_mean_: Average velocity of pupil constriction
PD: Pupil diameter
LSTM: Long short-term memory
CNN: Convolutional neural network
AUC: Area under the receiver operating characteristic curve

## References

[1] Li X, Chan E, Liao J, Wong T, Aung T, Cheng CY (2013). Number of people with glaucoma in Asia in 2020 and 2040: a hierarchical Bayesian meta-analysis. Invest Ophthalmol Vis Sci.

[2] Tham YC, Li X, Wong TY, Quigley HA, Aung T, Cheng CY (2014). Global prevalence of glaucoma and projections of glaucoma burden through 2040: a systematic review and meta-analysis. Ophthalmology.

[3] Su DH, Friedman DS, See JL, Chew PT, Chan YH, Nolan WP (2008). Degree of angle closure and extent of peripheral anterior synechiae: an anterior segment OCT study. Br J Ophthalmol.

[4] Mapstone R (1968). Mechanics of pupil block. Br J Ophthalmol.

[5] Foster PJ, Oen FT, Machin D, Ng TP, Devereux JG, Johnson GJ (2000). The prevalence of glaucoma in Chinese residents of Singapore: a cross-sectional population survey of the Tanjong Pagar district. Arch Ophthalmol.

[6] Sihota R, Ghate D, Mohan S, Gupta V, Pandey RM, Dada T (2008). Study of biometric parameters in family members of primary angle closure glaucoma patients. Eye (Lond).

[7] Fu H, Li F, Sun X, Cao X, Liao J, Orlando JI (2020). AGE challenge: angle closure glaucoma evaluation in anterior segment optical coherence tomography. Med Image Anal.

[8] Zheng C, Cheung CY, Narayanaswamy A, Ong SH, Perera SA, Baskaran M (2012). Pupil dynamics in Chinese subjects with angle closure. Graefes Arch Clin Exp Ophthalmol.

[9] Quigley HA, Silver DM, Friedman DS, He M, Plyler RJ, Eberhart CG (2009). Iris cross-sectional area decreases with pupil dilation and its dynamic behavior is a risk factor in angle closure. J Glaucoma.

[10] Narayanaswamy A, Zheng C, Perera SA, Htoon HM, Friedman DS, Tun TA (2013). Variations in iris volume with physiologic mydriasis in subtypes of primary angle closure glaucoma. Invest Ophthalmol Vis Sci.

[11] Ganeshrao SB, Mani B, Ulganathan S, Shantha B, Vijaya L (2012). Change in iris parameters with physiological mydriasis. Optom Vis Sci.

[12] Seager FE, Jefferys JL, Quigley HA (2014). Comparison of dynamic changes in anterior ocular structures examined with anterior segment optical coherence tomography in a cohort of various origins. Invest Ophthalmol Vis Sci.

[13] Aptel F, Denis P (2010). Optical coherence tomography quantitative analysis of iris volume changes after pharmacologic mydriasis. Ophthalmology.

[14] Quigley HA (2010). The iris is a sponge: a cause of angle closure. Ophthalmology.

[15] Lifton J, Burkemper B, Jiang X, Pardeshi AA, Richter G, McKean-Cowdin R (2022). Ocular biometric determinants of dark-to-light change in angle width: the Chinese American eye study. Am J Ophthalmol.

[16] Baskaran M, Foo RC, Cheng CY, Narayanaswamy AK, Zheng YF, Wu R (2015). The prevalence and types of glaucoma in an urban Chinese population: the Singapore Chinese Eye Study. JAMA Ophthalmol.

[17] Zheng C, Guzman CP, Cheung CY, He Y, Friedman DS, Ong SH (2013). Analysis of anterior segment dynamics using anterior segment optical coherence tomography before and after laser peripheral iridotomy. JAMA Ophthalmol.

[18] Hao H, Zhao Y, Yan Q, Higashita R, Zhang J, Zhao Y (2021). Angle-closure assessment in anterior segment OCT images via deep learning. Med Image Anal.

[19] Hao H, Fu H, Xu Y, Yang J, Li F, Zhang X, et al. Open-appositional-synechial anterior chamber angle classification in AS-OCT sequences. In: Lecture Notes in Computer Science (including subseries Lecture Notes in Artificial Intelligence and Lecture Notes in Bioinformatics). 2020;12265 LNCS:715–24.

[20] Li F, Yang Y, Sun X, Qiu Z, Zhang S, Tun TA (2022). Digital gonioscopy based on three-dimensional anterior segment optical coherence tomography: an international multicenter study. Ophthalmology.

[21] Foster PJ, Buhrmann R, Quigley HA, Johnson GJ (2002). The definition and classification of glaucoma in prevalence surveys. Br J Ophthalmol.

[22] Friedman DS, Gazzard G, Foster P, Devereux J, Broman A, Quigley H (2003). Ultrasonographic biomicroscopy, Scheimpflug photography, and novel provocative tests in contralateral eyes of Chinese patients initially seen with acute angle closure. Arch Ophthalmol.

[23] Zheng C, Cheung CY, Aung T, Narayanaswamy A, Ong SH, Friedman DS (2012). In vivo analysis of vectors involved in pupil constriction in Chinese subjects with angle closure. Invest Ophthalmol Vis Sci.

[24] Li M, Chen Y, Chen X, Zhu W, Chen X, Wang X (2018). Differences between fellow eyes of acute and chronic primary angle closure (glaucoma): an ultrasound biomicroscopy quantitative study. PLoS ONE.

[25] Zheng C, Xie X, Huang L, Chen B, Yang J, Lu J (2020). Detecting glaucoma based on spectral domain optical coherence tomography imaging of peripapillary retinal nerve fiber layer: a comparison study between hand-crafted features and deep learning model. Graefes Arch Clin Exp Ophthalmol.

[26] Liu S, Yu M, Ye C, Lam DSC, Leung CK (2011). Anterior chamber angle imaging with swept-source optical coherence tomography: an investigation on variability of angle measurement. Invest Ophthalmol Vis Sci.

[27] Zhang Y, Li SZ, Li L, He MG, Thomas R, Wang NL (2016). Dynamic iris changes as a risk factor in primary angle closure disease. Invest Ophthalmol Vis Sci.

[28] Woo EK, Pavlin CJ, Slomovic A, Taback N, Buys YM (1999). Ultrasound biomicroscopic quantitative analysis of light-dark changes associated with pupillary block. Am J Ophthalmol.

[29] Williams D, Zheng Y, Davey PG, Bao F, Shen M, Elsheikh A (2016). Reconstruction of 3D surface maps from anterior segment optical coherence tomography images using graph theory and genetic algorithms. Biomed Signal Process Control.

[30] He K, Zhang X, Ren S, Sun J. Deep residual learning for image recognition. In: Proceedings of the IEEE Conference on Computer Vision and Pattern Recognition. 2016. p. 770–8.

[31] Hochreiter S, Schmidhuber J (1997). Long short-term memory. Neural Comput.

[32] Donahue J, Hendricks LA, Guadarrama S, Rohrbach M, Venugopalan S, Darrell T, et al. Long-term recurrent convolutional networks for visual recognition and description. In: Proceedings of the IEEE Computer Society Conference on Computer Vision and Pattern Recognition. 2015. p. 2625–34. 10.1109/TPAMI.2016.2599174 27608449

[33] Kay W, Carreira J, Simonyan K, Zhang B, Hillier C, Vijayanarasimhan S, et al. The kinetics human action video dataset. arXiv:1705.06950. 2017.

[34] Zhang Y, Hedo R, Rivera A, Rull R, Richardson S, Tu XM (2019). Post hoc power analysis: is it an informative and meaningful analysis?. Gen Psychiatr.

[35] Fu H, Baskaran M, Xu Y, Lin S, Wong DWK, Liu J (2019). A deep learning system for automated angle-closure detection in anterior segment optical coherence tomography images. Am J Ophthalmol.

[36] Xu Y, Liu J, Cheng J, Lee BH, Wong DWK, Baskaran M (2013). Automated anterior chamber angle localization and glaucoma type classification in OCT images. Annu Int Conf IEEE Eng Med Biol Soc.

[37] Xu Y, Liu J, Wong DWK, Baskaran M, Perera SA, Aung T. Similarity-weighted linear reconstruction of anterior chamber angles for glaucoma classification. In: 2016 IEEE 13th International Symposium on Biomedical Imaging (ISBI). IEEE; 2016. p. 693–7.

[38] Fu H, Xu Y, Lin S, Wong DWK, Mani B, Mahesh M, et al. Multi-context deep network for angle-closure glaucoma screening in anterior segment OCT. In: International Conference on Medical Image Computing and Computer-Assisted Intervention. Springer; 2018. p. 356–63.

[39] Tan M, Le QV. Efficientnet: rethinking model scaling for convolutional neural networks. arXiv preprint. arXiv:190511946. 2019.

[40] Nongpiur ME, Sakata LM, Friedman DS, He M, Chan YH, Lavanya R (2010). Novel association of smaller anterior chamber width with angle closure in Singaporeans. Ophthalmology.

[41] Fu H, Xu Y, Lin S, Wong DWK, Baskaran M, Mahesh M (2020). Angle-closure detection in anterior segment OCT based on multilevel deep network. IEEE Trans Cybern.

[42] Quigley HA (2009). Angle-closure glaucoma-simpler answers to complex mechanisms: LXVI Edward Jackson Memorial Lecture. Am J Ophthalmol.

[43] Leung CK, Cheung CY, Li H, Dorairaj S, Yiu CK, Wong AL (2007). Dynamic analysis of dark-light changes of the anterior chamber angle with anterior segment OCT. Invest Ophthalmol Vis Sci.

[44] Huang EC, Barocas VH (2004). Active iris mechanics and pupillary block: steady-state analysis and comparison with anatomical risk factors. Ann Biomed Eng.

