# Supplementary material for: Dynamic analysis of iris changes and a deep learning system for automated angle-closure classification based on AS-OCT videos
Source: Eye Vis (Lond). 2022 Nov 5;9:41. doi: 10.1186/s40662-022-00314-1 (PMC9636810; doi:10.1186/s40662-022-00314-1)
Supplement: Supplementary file 1 — Additional file 1. The specific categories for the subjects of the angle-closure group. [file 40662_2022_314_MOESM1_ESM.docx]

**Supplemental Text**

**Subjects**

Angle-closure group: the subjects included four categories: primary angle-closure suspect (PACS), primary angle-closure (PAC), primary angle-closure glaucoma (PACG) and fellow eyes of previous acute primary angle-closure (APAC) [1–4].

(1) PACS was defined as eyes in which at least 180 of the posterior pigmented trabecular meshwork was not visible on non-indentation gonioscopy (narrow angle) with intraocular pressure (IOP) less than or equal to 21 mmHg, healthy optic disc, and without peripheral anterior synechiae (PAS);

(2) PAC was defined as eyes with narrow angles, healthy optic discs, and visual fields, but with elevated IOP (defined as an IOP > 21 mmHg), and/or PAS;

(3) PACG was defined as eyes with narrow angles accompanied with IOP greater than 21 mmHg, and glaucomatous optic neuropathy (cup/disc ratio greater than or equal to 0.7, or binocular cup/disc ratio asymmetry greater than 0.2 or local disc edge atrophy), as well as visual field impairment (abnormal glaucoma semi-visual field examination, abnormal model standard deviation *P* < 0.05, or at least three adjacent sites *P* < 0.05 in the pattern bias probability map, of which one site *P* < 0.01. The fixed loss rate was < 20%, false positive rate was < 33%, false-negative rate was < 33%). For these subjects, there existed reflection to light [5].

(4) Fellow eyes of previous APAC: APAC was defined as the presence of at least two of the following symptoms: (1) eye pain, or periocular pain, nausea and vomiting, transient blurred vision; (2) a presenting IOP of more than 28 mmHg on Goldmann applanation tonometry; (3) at least three of the following signs appeared: conjunctival congestion, corneal epithelial edema, mid-dilated nonreactive pupil, shallow anterior chamber. Additionally, the contralateral eye had no attack and had the anatomical characteristics of angle-closure glaucoma: shallow anterior chamber, narrow chamber angle, and the presence of light reflection [1,3].

**Supplemental Reference**

1. Zheng C, Cheung CY, Aung T, Narayanaswamy A, Ong SH, Friedman DS, et al. In vivo analysis of vectors involved in pupil constriction in Chinese subjects with angle closure. Invest Ophthalmol Vis Sci. 2012;53(11):6756–62.

2. Friedman DS, Gazzard G, Foster P, Devereux J, Broman A, Quigley H, et al. Ultrasonographic biomicroscopy, Scheimpflug photography, and novel provocative tests in contralateral eyes of Chinese patients initially seen with acute angle closure. Arch Ophthalmol. 2003;121(5):633–42.

3. Zheng C, Cheung CY, Narayanaswamy A, Ong SH, Perera SA, Baskaran M, et al. Pupil dynamics in Chinese subjects with angle closure. Graefes Arch Clin Exp Ophthalmol. 2012;250(9):1353–9.

4. Li M, Chen Y, Chen X, Zhu W, Chen X, Wang X, et al. Differences between fellow eyes of acute and chronic primary angle closure (glaucoma): an ultrasound biomicroscopy quantitative study. PLoS One. 2018;13(2):e0193006.

5. Zhang Y, Li SZ, Li L, He MG, Thomas R, Wang NL. Dynamic iris changes as a risk factor in primary angle closure disease. Invest Ophthalmol Vis Sci. 2016;57(1):218–26.
